# Congenital syphilis in Brazil: distribution of cases notified from 2009 to 2016

**DOI:** 10.1590/0037-8682-0338-2020

**Published:** 2020-11-25

**Authors:** Adriana Sousa Rêgo, Luciana Cavalcante Costa, Liliane dos Santos Rodrigues, Ricardo Amorim de Sousa Garcia, Flor de Maria Araújo Mendonça Silva, Aurean D’eça, Livia dos Santos Rodrigues

**Affiliations:** 1 Centro Universitário do Maranhão, Programa de Pós-Graduação em Gestão de Programas e Serviços de Saúde, São Luís, MA, Brasil.; 2 Universidade Federal do Maranhão, Centro de Pesquisas, Departamento de Saúde Pública, São Luís, MA, Brasil.; 3 Universidade Federal do Maranhão, Departamento de Biologia, São Luís, MA, Brasil.; 4 Universidade Federal do Maranhão, Departamento de Enfermagem, São Luís, MA, Brasil.; 5 Universidade de São Paulo, Faculdade de Medicina de Ribeirão Preto, Departamento de Puericultura e Pediatria, Ribeirão Preto, SP, Brasil.

**Keywords:** Congenital Syphilis, Time Series Studies, Public Health

## Abstract

**INTRODUCTION:**

Congenital syphilis is considered a severe public health problem because it accounts for approximately 40% of the perinatal mortality rates, 25% of stillbirths, and 14% of neonatal deaths, in addition to causing severe consequences for the fetus. This study aimed to describe the rates of congenital syphilis in children under one year of age in Brazilian capitals from 2009 to 2016.

**METHODS:**

Ecological time series study, using rates of congenital syphilis in children under one year of age and living in Brazilian capitals. The Prais-Winsten regression model was used to assess the trend.

**RESULTS:**

A total of 44,056 cases of congenital syphilis in children under one year of age were reported in Brazilian capitals between 2009 and 2016. The highest rate of congenital syphilis in children under one year of age occurred in 2016 in Porto Alegre (31.07/1,000 live births). The Northeastern capitals showed high rates, particularly the capital Recife (23.67/1,000 live births).

**CONCLUSIONS:**

Congenital syphilis represents a major challenge for public policies. The need for improvements in the quality of prenatal care is highlighted, as it is essential to reduce the alarming rates.

## INTRODUCTION

Syphilis, which is caused by the *Treponema pallidum* bacterium, is an infectious disease that can be transmitted through intimate contact, sexual intercourse, or during the gestational period (transmission from mother to child). This latter type of infection is referred to as congenital syphilis^1^ ^,^ ^2^, and may occur transplacentally or during delivery^3^.

Syphilis treatment is easy to access and simple to perform and therefore, it management during pregnancy should be straightforward. The diagnosis and treatment should be performed in a timely manner in the early period of pregnancy. However, being a sexually transmitted infection (STI) makes it a difficult-to-approach situation, especially when experienced in a sensitive moment (pregnancy). 

This implies that the quality of prenatal care is a key aspect for the diagnosis and a consequent improvement in syphilis control. This can be done with improvement in access, coverage, and quality of care at the primary level, and promotion of the right to the Venereal Disease Research Laboratory (VDRL) test in the first prenatal care consultation, in the third trimester of pregnancy, and at the time of hospitalization for childbirth. Another important point for reducing syphilis is the treatment of the partners, a procedure that prevents reinfection^4^. 

The severity of the congenital syphilis situation can be seen in the high number of reported cases. As shown in the Syphilis Epidemiological Bulletin (2019)^5^, between the years 2008 and 2018 there were 26,219 cases of congenital syphilis reported in Brazil; however, there was an increase of 5.2% in the number of cases observed from 2017 to 2018, with cases in the Northeast and Southeast regions being the most frequent. This increasing trend in the data was already present in the 2019 bulletin^5^.

A national study, with 23,894 mothers, carried out in 2011-2012 through hospital interviews, data from medical records, and prenatal cards, estimated an incidence of congenital syphilis of 3.51 per thousand live births (95% CI: 2.29-5.37) and a vertical transmission rate of 34.3% (95% CI: 24.7-45.4)^6^, varying from 1.35 per thousand in the Midwest Region to 4.03 per thousand in the Northeast Region^7^. 

The incidence rates of congenital syphilis in the state of Santa Catarina increased significantly (+1,190%). The same was seen in all the macro-regions in the studied period from 2007 to 2016, with predominance in children of Caucasian women with low schooling and under 20 years of age^8^. 

In the time trend and the spatial distribution of congenital syphilis in the state of Rio Grande do Sul (RS), of the 1,718,651 children born in the state between the years 2001 and 2012, 3,613 were notified and confirmed cases of CS^9^.

According to Furtado et al.^10^, the absence of treatment against congenital syphilis can have severe consequences for the baby, such as prematurity, abortion, and several types of sequelae. These sequelae vary according to the type of congenital syphilis.

Congenital syphilis is subdivided into two types. The first, referred to as precocious, occurs when the clinical manifestations of the disease occur between 0 and 2 years of age, with the following as its main examples: low birth weight, skin lesions, respiratory distress, anemia, jaundice, and pseudoparalysis of the limbs. In the second type, referred to as late, the child over 2 years of age may have an elevated palatal arch, neurological deafness, Clutton joints, and learning difficulties, among other health problems^11^.

Given the above, this study aimed to describe the rates of congenital syphilis in children under one year of age living in Brazilian capitals from 2009 to 2016.

## METHODS

This is a time series ecological study in which the rates of congenital syphilis were analyzed in children under one year of age residing in Brazilian capitals (Porto Velho, Rio Branco, Manaus, Boa Vista, Belém, Macapá, Palmas, São Luís, Teresina, Fortaleza, Natal, João Pessoa, Recife, Maceió, Aracaju, Salvador, Belo Horizonte, Vitória, Rio de Janeiro, São Paulo, Curitiba, Florianópolis, Porto Alegre, Campo Grande, Cuiabá, Goiânia, and Brasília). 

The descriptive analysis of the confirmed cases recorded the following parameters: prenatal care (yes or no), maternal syphilis (during prenatal care, at the time of delivery, after delivery, or not performed), treatment of the partner (yes or no), and evolution (alive, death due to the notified illness, or death due to another cause) from 2009 to 2016. 

Data collection was carried out through the information systems of the Informatics Department of the Unified Health System (DATASUS). To calculate the rate of congenital syphilis in children under one year of age, the number of confirmed cases by capital of residence in the period from 2009 to 2016 provided by the Notification Diseases Information System (*Sistema de Informação de Agravos de Notificação*, SINAN) was used as numerator and, as denominator, the number of live births to mothers living in the same place and period obtained from the Live Birth Information System (*Sistema de Informações sobre Nascidos Vivos*, SINASC), multiplied by one thousand. 

The analyses were performed using Stata 14.0 statistical software (StataCorp, Texas, USA), and the results were displayed through graphs and tables with relative and absolute frequencies. 

Since it used data in the public domain and in accordance with Resolution No. 466 of December 12^th^, 2012 of the National Health Council (*Conselho Nacional de Saúde*, CNS), this study was exempted from consideration by the Research Ethics Committee.

## RESULTS

A total of 44,056 congenital syphilis cases were reported in children under one year of age in Brazilian capitals from 2009 to 2016. In all the capitals, the majority of mothers attended prenatal care visits, especially in Boa Vista, with a percentage reaching 90.2%. In the Northeastern capitals, with the exception of Teresina, Rio Branco, Manaus, and Belém, the diagnosis of maternal syphilis occurred only at the time of delivery. Macapá was the only capital where the highest percentage of maternal syphilis diagnoses had occurred after delivery (37.2%). In the rest of the capitals (Porto Velho, Boa Vista, Palmas, and all those in the South and Southeast regions), the diagnosis of maternal syphilis occurred during prenatal care, with Curitiba standing out with the highest percentage (72.0%). In all the capital cities, in most cases, the treatment of the partner was not addressed, especially in Aracaju (94.4%) (Table 1).

**TABLE 1:** Characteristics of the notified cases of congenital syphilis in children under one year of age according to prenatal care, maternal syphilis, and treatment of the partner in Brazilian capitals from 2009 to 2016.

	Prenatal care				Maternal syphilis								Treatment of the partner			
Capital of residence	Yes		No		During prenatal care		At the time of delivery		After delivery		Not performed		Yes		No	
	N	%	N	%	N	%	N	%	N	%	N	%	N	%	N	%
Porto Velho	222	80.7	53	19.3	141	50.0	100	35.5	39	13.8	2	0.7	33	13.8	207	86.3
Rio Branco	167	76.6	51	23.4	72	33.8	115	54.0	24	11.3	2	0.9	24	11.3	189	88.7
Manaus	1,006	69.9	434	30.1	562	39.3	758	53.0	101	7.1	9	0.6	308	30.8	691	69.2
Boa Vista	165	90.2	18	9.8	84	45.2	83	44.6	17	9.1	2	1.1	35	20.2	138	79.8
Belém	385	69.9	166	30.1	168	30.7	273	49.9	105	19.2	1	0.2	76	17.9	349	82.1
Macapá	363	77.1	108	22.9	163	33.3	138	28.2	182	37.2	6	1.2	80	21.9	286	78.1
Palmas	208	85.2	36	14.8	129	52.4	107	43.5	8	3.3	2	0.8	29	13.2	190	86.8
São Luís	516	76.3	160	23.7	198	33.7	338	57.5	46	7.8	6	1.0	80	15.6	433	84.4
Teresina	554	80.1	138	19.9	336	48.6	277	40.1	72	10.4	6	0.9	175	26.6	484	73.4
Fortaleza	3,387	74.0	1.191	26.0	2,123	46.6	2,143	47.0	266	5.8	28	0.6	626	15.3	3,459	84.7
Natal	794	80.4	194	19.6	420	42.1	506	50.8	68	6.8	3	0.3	184	22.6	631	77.4
João Pessoa	370	79.2	97	20.8	163	35.0	256	54.9	42	9.0	5	1.1	90	22.3	313	77.7
Recife	1,991	78.8	535	21.2	983	36.2	1,548	57.0	168	6.2	15	0.6	279	14.7	1,621	85.3
Maceió	945	78.7	256	21.3	360	26.8	773	57.5	210	15.6	2	0.1	107	9.5	1,023	90.5
Aracaju	341	55.9	269	44.1	220	35.3	366	58.7	37	5.9	0	0.0	34	5.6	578	94.4
Salvador	1,328	74.3	459	25.7	945	43.9	965	44.8	229	10.6	14	0.7	279	18.8	1,205	81.2
Belo Horizonte	1,121	85.8	185	14.2	900	69.1	319	24.5	74	5.7	9	0.7	173	17.6	812	82.4
Vitória	261	84.2	49	15.8	210	67.7	77	24.8	23	7.4	0	0.0	33	11.7	249	88.3
Rio de Janeiro	7,650	82.4	1.635	17.6	4,753	50.3	4.146	43.9	500	5.3	53	0.6	1,298	18.0	5,912	82.0
São Paulo	4,903	72.2	1.891	27.8	3,751	54.5	2,871	41.7	225	3.3	36	0.5	1,183	19.4	4,908	80.6
Curitiba	686	84.7	124	15.3	584	72.0	182	22.4	42	5.2	3	0.4	96	12.8	656	87.2
Florianópolis	202	86.3	32	13.7	156	62.2	76	30.3	18	7.2	1	0.4	54	24.5	166	75.5
Porto Alegre	2,209	77.9	625	22.1	1,724	62.6	915	33.2	110	4.0	7	0.3	336	18.3	1,496	81.7
Campo Grande	501	82.3	108	17.7	366	59.3	179	29.0	71	11.5	1	0.2	76	13.4	491	86.6
Cuiabá	250	76.5	77	23.5	173	51.3	133	39.5	26	7.7	5	1.5	26	11.7	197	88.3
Goiânia	198	65.8	103	34.2	173	55.1	94	29.9	43	13.7	4	1.3	62	23.3	204	76.7
Brasília	983	84.3	183	15.7	699	60.9	329	28.7	110	9.6	10	0.9	247	23.9	786	76.1

**FIGURE 1:**
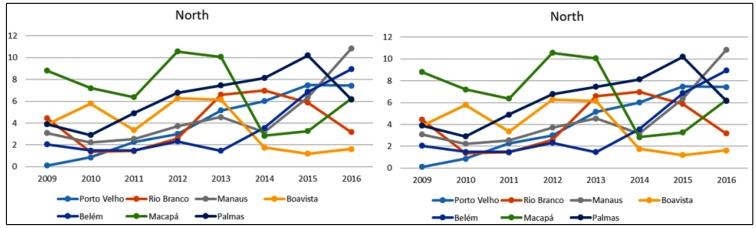
Distribution of congenital syphilis rates* in children under one year of age living in the capitals of the North and Northeast regions of Brazil from 2009 to 2016. *Per 1000 live births.

**FIGURE 2:**
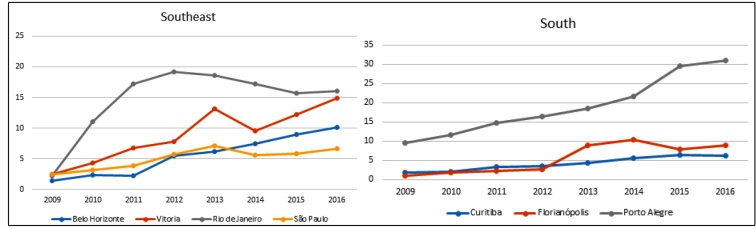
Distribution of congenital syphilis rates* in children under one year of age living in the capitals of the Southeast and South regions of Brazil from 2009 to 2016. *Per 1000 live births.

**FIGURE 3:**
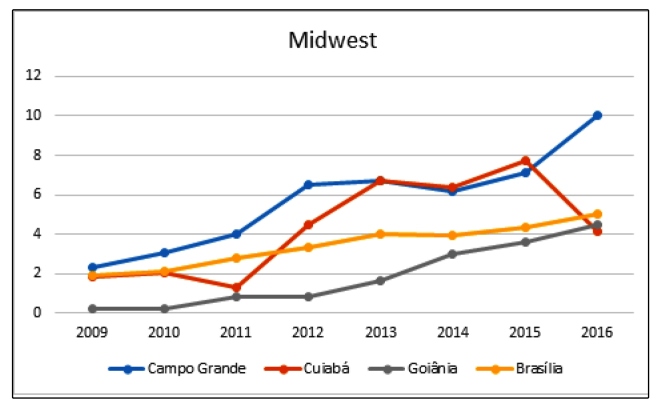
Distribution of congenital syphilis rates* in children under one year of age living in the capitals of the Midwest region of Brazil from 2009 to 2016. *Per 1000 live births.

The biggest oscillations, by region, were observed in the following capitals: Manaus (North), from 3.10/1000 live births in 2009 to 10.83/1000 live births 2016 (Table 2 and Figure 1); Teresina (Northeast), from 0.22/1000 live births in 2009 to 6.31/1000 live births in 2015 (it is important to highlight that the Northeastern capitals presented high rates, especially Recife, with 23.67/1000 live births) (Table 2 and Figure 1); Rio de Janeiro (Southeast), from 2.29/1000 live births in 2009, reaching 19.14/1000 live births in 2012 and 16.07/1000 live births in 2015 (Table 2 and Figure 2); Porto Alegre (South), from 9.48/1000 live births in 2009 to 31.07/1000 live births in 2016, the highest rate of congenital syphilis in the studied historical series (Table 2 and Figure 2); and Campo Grande (Midwest), from 2.34/1000 live births in 2009 to 10.05/1000 live births in 2016 (Table 2 and Figure 3).

**TABLE 2:** Congenital syphilis rates in children under one year of age in Brazilian capitals from 2009 to 2016.

Capital of residence	2009	2010	2011	2012	2013	2014	2015	2016
North								
Porto Velho	0.13	0.86	2.28	3.01	5.15	6.02	7.47	7.43
Rio Branco	4.43	1.39	1.44	2.60	6.60	6.98	5.88	3.17
Manaus	3.10	2.23	2.54	3.71	4.56	3.15	6.37	10.83
Boavista	3.89	5.79	3.35	6.29	6.14	1.77	1.19	1.62
Belém	2.06	1.49	1.50	2.33	1.46	3.57	6.87	8.93
Macapá	8.81	7.20	6.38	10.56	10.06	2.85	3.26	6.22
Palmas	3.91	2.91	4.92	6.80	7.45	8.13	10.19	6.17
Northeast								
São Luís	1.74	1.15	3.11	3.986	7.32	6.41	9.76	8.03
Teresina	0.22	1.18	1.79	3.330	5.91	6.67	16.31	15.36
Fortaleza	12.61	12.07	14.12	16.39	16.59	17.59	16.85	19.43
Natal	8.83	7.62	8.93	11.21	9.03	10.11	16.83	15.28
Joao Pessoa	2.27	2.26	3.86	6.81	5.71	8.82	10.73	0.93
Recife	8.11	9.95	13.36	12.37	18.38	19.56	23.15	23.67
Maceió	4.93	7.56	11.12	14.92	14.82	14.91	12.72	11.05
Aracaju	4.29	5.12	6.36	12.69	10.45	10.84	7.34	9.02
Salvador	1.02	2.58	5.08	7.61	11.21	11.26	13.07	16.76
Southeast								
Belo Horizonte	1.39	2.34	2.25	5.50	6.15	7.43	9.00	10.08
Vitoria	2.47	4.37	6.75	7.77	13.09	9.57	12.16	14.87
Rio de Janeiro	2.29	11.04	17.18	19.14	18.54	17.17	15.65	16.07
São Paulo	2.42	3.13	3.84	5.69	7.07	5.61	5.79	6.68
South								
Curitiba	1.72	2.09	3.32	3.54	4.29	5.52	6.36	6.24
Florianópolis	0.95	1.88	2.20	2.72	8.88	10.32	7.82	8.98
Porto Alegre	9.48	11.62	14.69	16.33	18.49	21.73	29.65	31.07
Midwest								
Campo Grande	2.34	3.04	3.98	6.49	6.71	6.19	7.11	10.05
Cuiabá	1.81	2.03	1.30	4.49	6.71	6.35	7.76	4.15
Goiânia	0.20	0.19	0.82	0.84	1.63	3.00	3.62	4.49
Brasília	1.88	2.10	2.76	3.33	3.97	3.91	4.37	5.02

## DISCUSSION

This study highlights the performance of prenatal care with satisfactory percentages in all Brazilian capitals, especially when it comes to the diagnosis of maternal syphilis. However, the quality of prenatal care can still be improved in a minority of capitals where the diagnosis of maternal syphilis was made only at the time of delivery or postpartum. It is also important to emphasize that not treating the partner of the woman identified as having syphilis persists as a public health problem.

According to information from the Ministry of Health, the notification of cases of congenital syphilis has generally grown in Brazil, especially since 2010. In 2006 the rate was 2.0 cases/thousand live births, and in 2015, it rose to 6.5 cases/thousand live births^12^, which is well beyond the maximum incidence rate indicated by the Pan American Health Organization (PAHO), which is 0.5 cases/thousand live births^13^.

The reason for the growth in the number of cases over the years is ambiguous. A number of studies assume that the increase in these numbers may not necessarily be associated merely with the increase in cases, but rather, be related to improvements in the records and in the notification system, training of health professionals, expansion of prenatal coverage due to the implementation of the Family Health Strategy health teams, and an increase in the effective work of the epidemiological surveillance programs^14^.

Implemented in 2011 by Ordinance No. 1,459 of June 24, the Ministry of Health Program - Rede Cegonha aims to structure and reorganize Brazilian maternal and child healthcare by adopting significant strategic measures such as facilitating access to Basic Health Units for rapid tests, continuous training for primary care professionals, and improvement of information systems records^15^.

Horta et al.^16^ attributed the increasing rates of congenital syphilis to inadequate or nonexistent treatment for the disease during pregnancy. It was found that 43% of pregnant women attended six or more prenatal consultations; however, only 3% underwent a VDRL in the 1^st^ trimester and another in the 3^rd^ trimester of their pregnancies, which suggests failures in the prevention and control of syphilis on the part of the health professionals^17^. A survey carried out between 2007 and 2013 showed that the treatment for syphilis during pregnancy was considered inadequate or incomplete in 64.8% of cases^18^. 

A descriptive study carried out in Rondônia from 2009 to 2014 found that 60.1% of the mothers learned about the diagnosis of congenital syphilis during prenatal care, and 28.28% only at the time of delivery or curettage^19^. A study carried out in Rio de Janeiro from 2011 to 2014 showed that 43% of the women were diagnosed with syphilis during prenatal care and 44% during childbirth/curettage^20^. This high rate of diagnosis in the postpartum period may be caused by the failure to perform the VDLR test, which the Ministry of Health recommends to be performed in the 1^st^ and 3^rd^ trimesters of pregnancy. Therefore, congenital syphilis is still a neglected disease despite all the protocols recommended by the Ministry of Health^21^.

The identification of pregnant women with syphilis must be done early so that there is a quick and effective decision making regarding the treatment. To identify and elaborate actions to control gestational syphilis, the PAHO and the World Health Organization (WHO) suggest monitoring indicators aimed at reducing transmission to the fetus, thereby avoiding negative outcomes. Among the proposed indicators are the following: pregnant women attending at least one prenatal visit, conducting the tests for syphilis, and for those infected, receiving at least one dose of benzathine penicillin^22^ ^,^ ^23^.

In Brazil, syphilis is more prevalent in pregnant women who do not attend prenatal care^24^, in those with greater social vulnerability^25^, and in those residing in locations with difficult accessibility to syphilis testing laboratories^26^. 

Modeling studies indicate that an important element in attaining the goal of eliminating congenital syphilis would be the treatment of all sexual partners of women diagnosed with syphilis. According to data from SINAN^27^, in 2015 only 13.9% of the sexual partners received treatment for syphilis. It is noteworthy that treatment of the partner is fundamental to avoid reinfection in the pregnant woman, and failure to perform this treatment, or the performance of an inadequate treatment, is one of the criteria adopted by the Ministry of Health to define a case of congenital syphilis^20^.

It can be understood based on studies^28^ that the expansion with quality of care in the Family Health Strategy and adherence to the Cegonha Network are elements that contributed to the improvement of notification and, consequently, the real increase in the number of cases of congenital syphilis in Brazil. However, some obstacles, such as unprotected sexual practices, are challenging in interrupting the congenital syphilis transmission chain^29^.

The complexity of the diagnosis of congenital syphilis in newborns (NBs) infected with *T. pallidum* must also be considered. The infected NB, in most cases, is asymptomatic at birth, the VDRL titers may be much lower compared to the maternal titration at this stage of life and the deficiency of the appropriate test for diagnosis in the public health network (CSF test)^29^. This denotes the fragility of the system, underreporting, late diagnosis, and, consequently, more severe complications to the child.

This study had limitations due to the use of secondary data in the public domain, with the possibility of underreported data, implying that the incidence rates may be higher than the ones shown. Another limitation that reduced the comparison power of the findings was the scarcity of reported data regarding the distribution of congenital syphilis at the national level over time. However, the SINAN is an official system with robust information and is widely used in scientific research studies, which bring important results to support the planning of public policies and effective changes in the epidemiological scenario of a given region and even the country.
